# Emerging Mechanistic Insights into AAA Complexes Regulating Proteasomal Degradation

**DOI:** 10.3390/biom4030774

**Published:** 2014-08-06

**Authors:** Friedrich Förster, Jan M. Schuller, Pia Unverdorben, Antje Aufderheide

**Affiliations:** Department of Molecular Structural Biology, Max-Planck Institute of Biochemistry, Martinsried D-82152, Germany; E-Mails: janschu@biochem.mpg.de (J.M.S.); unverdor@biochem.mpg.de (P.U.); aufderhe@biochem.mpg.de (A.A.)

**Keywords:** AAA-ATPases, 26S proteasome, Cdc48, p97, unfoldase, segregase, cryo-EM

## Abstract

The 26S proteasome is an integral element of the ubiquitin-proteasome system (UPS) and, as such, responsible for regulated degradation of proteins in eukaryotic cells. It consists of the core particle, which catalyzes the proteolysis of substrates into small peptides, and the regulatory particle, which ensures specificity for a broad range of substrates. The heart of the regulatory particle is an AAA-ATPase unfoldase, which is surrounded by non-ATPase subunits enabling substrate recognition and processing. Cryo-EM-based studies revealed the molecular architecture of the 26S proteasome and its conformational rearrangements, providing insights into substrate recognition, commitment, deubiquitylation and unfolding. The cytosol proteasomal degradation of polyubiquitylated substrates is tuned by various associating cofactors, including deubiquitylating enzymes, ubiquitin ligases, shuttling ubiquitin receptors and the AAA-ATPase Cdc48/p97. Cdc48/p97 and its cofactors function upstream of the 26S proteasome, and their modular organization exhibits some striking analogies to the regulatory particle. In archaea PAN, the closest regulatory particle homolog and Cdc48 even have overlapping functions, underscoring their intricate relationship. Here, we review recent insights into the structure and dynamics of the 26S proteasome and its associated machinery, as well as our current structural knowledge on the Cdc48/p97 and its cofactors that function in the ubiquitin-proteasome system (UPS).

## 1. Introduction

The ubiquitin-proteasome system (UPS) is responsible for the regulated degradation of proteins in eukaryotic cells [1,2]. Proteins that are to be removed at a specific state of a cell, as well as proteins that do not meet the cellular quality criteria (e.g., folding, glycosylation, integration into their predestined complexes) are labeled with polyubiquitin (poly-Ub) chains by a cascade of E1, E2 and E3 enzymes; ubiquitin (Ub) moieties are attached to lysines of the substrate, which are then ubiquitylated themselves at their lysine residues, giving rise to different types of poly-Ub chains. The 26S proteasome degrades the polyubiquitylated substrates into short peptides in an ATP-dependent manner. The UPS is essential in all eukaryotic cells, making it an important drug target for diseases, including cancer [3] and neurodegenerative diseases [4].

Across organisms, the UPS primarily varies most upstream: higher eukaryotes possess a much higher number of E3 Ub ligases (*Homo sapiens*: >600 [5,6] *vs*. *Saccharomyces cerevisiae* 60–100 [7]) and E2 conjugating enzymes (37 *vs*. 11) that specialize in the ubiquitylation of selected substrates, whereas the downstream machinery is largely identical in all organisms. In particular, the 26S proteasome is functionally and structurally highly conserved [8]. Analogous to prokaryotic ATP-dependent proteases (reviewed in [9]), its central element is a cylindrical core particle (CP), also referred to as the 20S proteasome, which is responsible for the proteolytic cleavage of substrates. The CP itself is a rather unspecific protease, and narrow pores at both cylinder ends prevent uncontrolled access of substrates to the active sites in the inner compartment of the CP. Specificity is conferred by the 19S regulatory particle (RP), which binds to one or both cylinder ends of the CP. The RP recruits polyubiquitylated substrates and prepares them for degradation. It consists of a heterohexameric ATPase associated with the diverse cellular activities (AAA) module (subunits Rpt1-6) (reviewed in [10]) and 13 non-ATPases (Rpn1-3, 5-13, 15), which assemble around the AAA-module. The primary functions of the Rpns seem to be substrate recruitment and deubiquitylation of substrates prior to degradation, whereas the AAA-ATPase module exerts the force that is required for substrate unfolding and translocation into the CP. In addition to the Rpts and Rpns, a number of cofactors, commonly referred to as proteasome-interacting proteins (PIPs), is more loosely associated with the 26S proteasome, including deubiquitylating enzymes, shuttling Ub receptors and E3 ligases, which all regulate proteasomal function in the cell.

Polyubiquitylation alone by the E1/E2/E3 machinery, however, is often not sufficient for the degradation of substrates by the 26S proteasome. This holds in particular for the broad range of substrates targeted by the protein quality control machineries [11]. Another AAA-ATPase complex, called cell division cycle protein 48 (Cdc48) in yeast and p97 in mammals (in the past, also often referred to as VCP), has a central role as a facilitator of proteasomal degradation [12,13]. In conjunction with various cofactors, Cdc48/p97 promotes poly-Ub chain elongation, segregates substrates from interactors and escorts them to the 26S proteasome.

In this review, we focus on recent structural and mechanistic insights into the 26S proteasome and Cdc48, including their associated cofactors. First, we briefly summarize our knowledge on the structure of the CP and its ATP-dependent regulators in prokaryotes before giving an account of the structure of the eukaryotic RP and the conformational changes that facilitate its function, as well as our current understanding of the major PIPs. We then recapitulate recent information on the Cdc48 machinery and discuss analogies of this machinery to the RP.

## 2. Core Particle

Archaea possess a CP that is simpler than the eukaryotic CP. Due to the comparably straightforward recombinant expression of the *Thermoplasma acidophilum* CP, the first atomic structure was solved for this species [14]. The archaeal CP consists of four stacked homoheptameric rings in the order αββα. Both types of subunits, α and β, are members of the N-terminal nucleophile hydrolase superfamily, but the α-subunits possess an N-terminal extension, rendering them proteolytically inactive. The catalytically active sites are positioned in the cavity formed by the inner β-subunits, whereas the “antechambers” formed by the α- and β-subunits are responsible for maintaining substrates in an unfolded state prior to their degradation [15]. The passage through the center of the α-ring (the “gate”) is regulated by the highly dynamic N-terminal tails of the α-subunits, allowing only unfolded substrates to enter [16,17,18,19].

While the eukaryotic CP is assembled of heteroheptamers (subunits α_1_–α_7_ and β_1_–β_7_, respectively) instead of homoheptamers, the overall structure of the holocomplex is highly conserved [20]. Of the seven eukaryotic β-subunits, only β_1_, β_2_ and β_5_ are catalytically active. The archaeal β-subunits mostly show chymotrypsin-like peptidase activity, whereas the eukaryotic β_1_, β_2_ and β_5_ subunits have caspase-, trypsin- and chymotrypsin-like activity, respectively. The active sites can be inhibited by a number of chemical compounds, including the anti-cancer drug bortezomib (reviewed in [21]). In mammals, different orthologs of the three catalytically active subunits have evolved, which are expressed in specific tissues and give rise to “immunoproteasomes” (iCPs) and “thymoproteasomes” (tCPs), in addition to constitutive CPs (reviewed in [22]). The crystal structure of the iCP suggests that stabilization of a proteolytic transition state of β_5_ is mostly responsible for the enhanced major histocompatibility complex class I (MHC-I) antigen generation by iCPs [23].

## 3. ATP-Dependent Regulators of the Archaeal Core Particle

Among bacteria, actinomycetes exhibit a UPS-related degradation system based on the prokaryotic Ub-like protein (PUP) [24,25]. More recently, it has become evident that also archaea possess a system similar to the UPS that makes use of small archaeal modifier proteins (SAMPs), which are Ub homologs [26]. Since the archaeal system is more closely related to the UPS, we restrict ourselves to the discussion of this simplified UPS cousin. The Ub-activating E1 enzyme homolog UbaA accomplishes the ligation of SAMP to lysine residues of substrates (“sampylation”) [26,27]. The reverse process, desampylation, is achieved by enzymes that are similar to JAB1/MPN/MOV34 (JAMM) deubiquitylating enzymes (DUBs) [28]. DUBs of this class, including Rpn11, possess an Mpr1-Pad1 N-terminal (MPN) domain with a characteristic JAMM motif that gives rise to metalloprotease activity [29].

Many archaeal organisms have a gene that codes for the proteasome activating nucleotidase (PAN), which has high sequence similarity to the Rpts and forms homohexamers [30]. Indeed, molecular studies have shown that PAN promotes degradation of small peptides by the CP in a similar manner as the RP does, and it forms complexes with the CP that get stabilized in the presence of ATP-γS [31]. PAN’s role as a homolog of the proteasomal proteases is also underscored by the structures of PAN fragments, which are highly similar to their eukaryotic counterparts [32,33]; similar to the Rpts, PAN assembles to a trimer of dimers with N-terminal coiled coil dimers protruding from the pseudo six-fold symmetrical oligosaccharide binding fold (OB) ring, which resides on top of a ring formed by the AAA-domains (AAA-ring).

Pivotal for the binding of PAN to the CP is a C-terminal motif comprising a hydrophobic residue (Hb) and a tyrosine followed by a residue of any type (HbYX) [34]. Cryo-EM studies of small HbYX containing peptides bound to the CP revealed that these motifs insert into pockets at the interfaces of the α-subunits and trigger substantial conformational changes of the CP [35]. The precise binding mode of the C-terminal peptides is still under debate, because the crystal structures of chimeric constructs yielded inconsistent results [36,37]. Among the Rpts, only Rpt2, Rpt3 and Rpt5 exhibit the HbYX motif at their C-termini [34]. These subunits bind to the CP in a similar manner as observed for isolated peptides, although the conformational changes of the CP were not seen in the holocomplex [38,39,40]. The reason for this discrepancy may be that the addition of isolated HbYX peptides results in the occupancy of all CP binding sites, whereas only a fraction of pockets is engaged in the CP-AAA-ATPase complexes, due to the symmetry mismatch of CP and the AAA-ATPase module (pseudo-six-fold *vs*. pseudo-seven-fold). The accordingly more dynamic binding of the C-termini [41] likely results in more subtle effects on the free energy landscape of the CP gate that may, for example, enable easier gate opening upon peptide translocation.

For a long time, it has been puzzling that some archaea, like *T*. *acidophilum*, do not have a PAN gene, whereas the CP is strictly conserved [42]. Among the archaeal AAA-ATPases, Cdc48 homologs, originally coined VCP-like ATPase of *Thermoplasma acidophilum* (VAT) [43], and a group of proteins only found in *Archaeoglobus* and methanogenic archaea (AMA) also exhibit the C-terminal HbYX motif [44]. Thus, early on, both proteins were genuine candidates for CP regulators. However, it has only been shown recently that Cdc48 and AMA proteins can also bind the CP [45,46,47]. These studies all required stabilizing the CP-AAA-ATPase interaction using an ATPase mutation or cysteine cross-linking. Accordingly, PAN, Cdc48 and AMA proteins seem to have overlapping functions in archaea.

## 4. Molecular Architecture of the 19S Regulatory Particle

The RP comprises two independently assembling sub-complexes, the base and the lid [48]. The base consists of the Rpts, Rpn1, Rpn2 and Rpn13, whereas the lid comprises the remaining Rpns with the exception of Rpn10, which is apparently associated only after the base and the lid form the RP. The Rpts are arranged into a ring of the order Rpt1/2/6/3/4/5 [49,50], which adopts a specific register on the CP, as determined by protein interaction studies and chemical cross-linking [49,41,51]. However, due to the symmetry mismatch between the ATPase module and CP, the binding between both complexes is not tight and allows for considerable lateral motion of the AAA-ATPase hexamer on the CP (see below). Rpn1 and Rpn2, the two largest, evolutionarily-related Rpns, bind to the tips of the coiled coil dimers of Rpt1/2 and Rpt3/6, respectively [38,39,40,52]. Whereas Rpn1 is positioned as somewhat isolated in the 26S holocomplex, reflecting its role as a docking platform for many PIPs, Rpn2 is tightly integrated with the lid, and the Ub receptor Rpn13 is positioned near the N-terminus of Rpn2 in the very periphery of the 26S holocomplex [53].

The lid subunits, Rpn9, 5, 6, 7, 3 and 12, share a similar architecture; their hallmarks are proteasome-COP9-initiation factor 3 (PCI) modules, which assemble into a heterohexameric horseshoe [39,40,54]. This horseshoe flanks the AAA-ATPase module and directly binds to the CP via Rpn6 and Rpn5, stabilizing the 26S proteasome during conformational switching [52]. The smallest Rpn, Rpn15 (also called Sem1 in yeast and Dss1 in mammals), stabilizes the horseshoe between Rpn7 and Rpn3 [55,56]. The C-termini of the PCI subunits together with those of the MPN domain-containing subunits, Rpn8 and Rpn11, assemble into a helical bundle, which tethers the lid together [38,57]. Rpn8 and Rpn11 dimerize through their MPN domains [38,58,59]. The JAMM motif is absent in Rpn8, rendering this subunit catalytically inactive. In the 26S holocomplex, the DUB Rpn11 is positioned directly above the central pore of the ATPase module, enabling it to remove the poly-Ub chain from the substrate immediately prior to degradation; *i*.*e*., proteolytic cleavage occurs between the ubiquitylated lysine of the substrate and the poly-Ub molecule [60,61]. For a more detailed review of the RP architecture, we refer to [62].

## 5. Conformational Switching of the 26S Proteasome

In all initial high-resolution cryo-EM studies, the 26S proteasome was imaged in the presence of ATP and in the absence of substrate [38,40,51,63,64]. Although, under these conditions, the AAA-ATPase subunits may continuously exchange nucleotides, relatively well-defined structures were obtained, indicating that the particles were predominantly in a single conformation. A hallmark of the AAA-ATPase configuration in this predominant low-energy state is a staircase- or lock washer-like arrangement of the AAA-domains [39], similar to that observed in V-ATPases [65], RecA/DnaB-type helicases [66] and AAA-type helicases [67].

**Figure 1:**
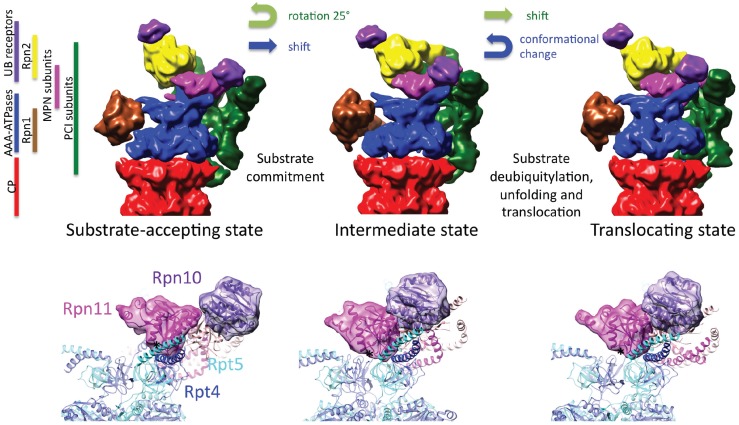
Conformational states of the 26S proteasome. (**Top row**) The *S*. *cerevisiae* 26S proteasome has been imaged in three distinct conformational states using cryo-EM single particle analysis [52]. The EM densities are segmented according to their main functional modules indicated in the legend. Upon transition from the low-energy conformation (**left**) to the intermediate conformation (**center**), the non-ATPases rotate *en bloc* by approximately 25°, and the ATPase module shifts by approximately 5 Å. The ATPases undergo a large-scale conformational change during the transition of the 26S proteasome from the intermediate to the high-energy conformation (**right**) concomitant with a shift of the non-ATPases. The hypothesized functions of the states are substrate recruitment (low-energy), substrate commitment (intermediate) and substrate translocation into the core particle (CP, high-energy). (**Bottom** **row**) Among the three states, the local environment of Rpn11 is essentially identical in the intermediate and translocating state, suggesting that the enzyme is active in both states.

Different approaches to stabilize alternative 26S conformations were the alteration of the free energy landscape by replacing ATP by ATP-γS in the buffer [68] and the addition of polyubiquitylated substrate to proteasomes with dysfunctional Rpn11 [69]. Somewhat surprisingly, both approaches revealed essentially the same conformational change: the AAA-ATPase hexamer undergoes a dramatic rearrangement concomitant with a ~20° rotation of the non-ATPases. In-depth classification of 26S proteasome particles in the presence of ATP and ATP-γS revealed a third conformational state, which is intermediate between the presumable low- and high-energy states [52]; the AAA-ATPase module essentially remains in the low-energy conformation, whereas the non-ATPases are positioned similar as in the high-energy state. These three states could facilitate the degradation of polyubiquitylated substrates in the following manner (Figure 1): Substrates primarily associate with the 26S proteasome when it adopts its low-energy conformation. In this state, substrate binding is still reversible, and proteasome-associated DUBs may process the poly-Ub chain (reviewed in [70]). Conformational switching to the intermediate conformation, possibly facilitated by different nucleotide loading of one site, may transfer the substrate to the mouth of the OB ring, committing the substrate to degradation [71,72,73]. In both, the intermediate and the high-energy state, the conformation of Rpn11 and its local environment seem to be essentially identical. The DUB is presumably active in these two states in contrast to the low-energy state; a composite active site may be formed by Rpn11, the AAA-ATPase hexamer and Rpn2 [58]. Cleavage of the poly-Ub chain may thus occur in both states. The high-energy state then enables unfolding and translocation of the substrate into the CP by releasing its energy, probably in rapid bursts, as seen for bacterial ATP-dependent proteases [74,75].

## 6. Regulation of Proteasomal Degradation by Proteasome-Interacting Proteins

In the crowded environment of the cell, many PIPs bind to the 26S proteasome, which largely dissociate during the typical biochemical purification procedures of the 26S proteasomes, due to the dynamical nature of these interactions [76,77]. Three major groups are most abundant among PIPs that all modulate proteasome activity by different means (reviewed in [7,8]): (i) the “shuttling” Ub receptors, Rad23 (HR23a/b in mammals), Dsk2 (PLIC-1) and Ddi1, mediate the binding of ubiquitylated substrates to the proteasome [78,79,80,81]; (ii) DUBs trim Ub chains and may prevent them from degradation (reviewed in [70]) (the Ub C-terminal hydrolase, Uch37 (also referred to as UchL5 in mammals), and the Ub-specific protease, Ubp6 (Usp14), are the most abundant proteasome-associated DUBs); (iii) E3 Ub ligases, in particular the subclass of E4 ligases that specializes in extending existing Ub chains, antagonize DUB function and couple ubiquitylation to degradation when binding to the 26S proteasome. Nine Ub ligases have been proposed to interact with the proteasome (reviewed in [82]), of which the E4 enzyme, HECT Ub ligase 5 (Hul5, UBE3B/C in mammals), is the most abundant one in 26S proteasome purifications [83].

**Figure 2:**
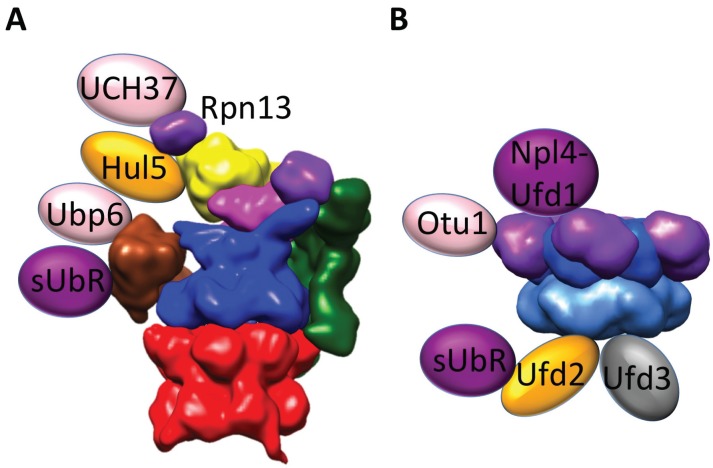
The most abundant cofactors of 26S proteasome and Cdc48 involved in the ubiquitin-proteasome system (UPS). (**A**) Proteasome-interacting proteins (PIPs). The DUB Ubp6 and all shuttling Ub receptors (sUbR), Rad23, Dsk2 and Ddi1, bind to Rpn1 via their UBL domains, whereas the E4-ligase Hul5 interacts with Rpn2. In higher eukaryotes, an additional DUB, UCH37, binds to the Ub receptor Rpn13. (**B**) In Cdc48, the majority of cofactors, including the heterodimeric Npl4-Ufd1 substrate recruiting cofactor and the DUB Out1, bind to the N-domain. Contrarily, the E4 Ligase, Ufd2, and the Ub-chain release factor, Ufd3, bind to the unstructured C-terminal tail. Additionally, the shuttling substrate receptors (sUbR), Rad23 and Dsk2, are associated via Ufd2.

**Figure 3:**
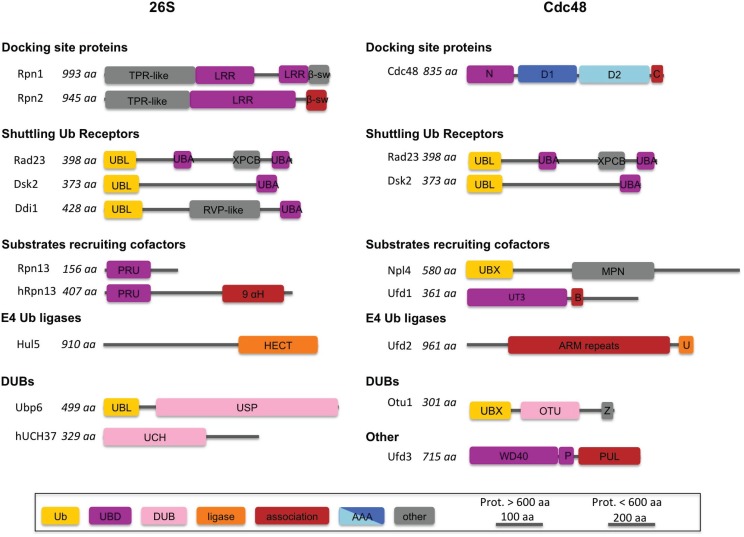
Schematic representation of sequences of PIPs and the major Cdc48-associated cofactors involved in the UPS. PIPs bind to the docking platforms, Rpn1 and Rpn2, all Cdc48-associated proteins to either the Cdc48 N-domain or the C-terminal tail (C). Yellow: domains with an Ub-related fold; purple: domains that bind to Ub and Ub-related domains (UBD); pink: DUB domains; orange: ubiquitin ligase domains; red: domains involved in PIP or Cdc48-cofactor association; blue: AAA-domain; gray: established domains with different or unknown functions. The following additional abbreviations are introduced: TPR: tetratricopeptide repeat; β-sw: β-sandwich domain; 9αH: alpha helical bundle; RVP: retroviral protease-like domain; XPCB: xeroderma pigmentosum group C protein-like domain; USP: ubiquitin-specific protease domain; B: BS1; ARM: armadillo-repeat containing domain; U: RING-like U-box domain; Z: Zn-finger motif; WD40: WD40-repeat containing domain; P: PLAA family ubiquitin binding (PFU) domain.

All currently established PIP associations involve Rpn1 and Rpn2 (Figure 2; for comprehensive reviews, we refer to [7,8,82,84]), and it has been suggested that these subunits coordinate PIP occupancy [85]. Essentially, all PIPs that are currently known to associate with Rpn1, including the shuttling Ub receptors and Ubp6, possess Ub-like (UBL) domains. *S*. *cerevisiae* Rad23, Dsk2 and Ddi1 all have been reported to bind to the toroid-shaped leucine-rich repeat (LRR) domain of Rpn1 via their UBL domains in a partially competitive manner (Figure 3) [81,86,87], whereas, in higher eukaryotes, they additionally bind to Ub receptors, in particular the Ub-interacting motifs of Rpn10 [88,89]. Likewise, Ubp6 associates with Rpn1 primarily via its UBL domain [83], albeit *in vitro* studies suggest that also other protein segments may contribute to the interaction [85]. A hallmark of Ubp6 is its activation upon binding to the 26S proteasome [83]. *Vice versa*, Ubp6 also influences proteasome activity: binding of Ub-conjugates and Ub-aldehyde to proteasome-bound Ubp6 accelerates degradation of short peptides [90], while Ubp6 delays proteasomal degradation of polyubiquitylated substrates independent of the Ubp6 isopeptidase activity [4,91].

The Ub receptor Rpn13, also referred to as Adrm1 in mammals, binds to the C-terminal domain of Rpn2 essentially in a stoichiometric ratio in *S*. *cerevisiae* (Figure 3) [39,53]. However, this “canonical” subunit binds dynamically in higher eukaryotes [76] and is typically found in substoichiometric amounts in 26S proteasome preparations [50,92]. Thus, Rpn13 might also be viewed as an Rpn2-associated PIP. Higher eukaryotes express an additional DUB, UCH37, which binds to a domain of Rpn13 that is only found in organisms with an UCH37 gene [92,93,94,95,96,97]. Furthermore, the E3/E4 ligase Hul5 avidly interacts with Rpn2 [98]. Hul5 and Ubp6 are suggested to be antagonists and cooperatively enable Ub chain remodeling [98]. The 26S proteasome structure would indeed allow binding of Ubp6 and Hul5 to Rpn1 and Rpn2, respectively, such that the proteins are positioned in mutual proximity (Figure 2). An additional function of proteasome-associated E2 and E3/E4 enzymes, as well as DUBs seems to be the (de)-ubiquitylation of 26S proteasome subunits regulating proteasomal activity [77]. The *in situ* arrangement of PIPs is a largely uncharted territory at this point. Further structural characterization will be essential for mechanistic understanding of their contribution to proteasomal regulation.

## 7. Cdc48/p97—A Facilitator of Proteasomal Degradation

Polyubiquitylation by the E1/E2/E3 machinery is not sufficient for proteasomal degradation of many substrates. The AAA-ATPase homohexamer cell division control protein 48 (Cdc48), commonly referred to as p97 and historically also as VCP in mammals, is a key facilitator of proteasomal degradation of many polyubiquitylated substrates in the cell. In particular, this protein complex is required for the extremely broad range of substrates that is degraded by different cellular quality control pathways: Cdc48 is a hallmark component of endoplasmic reticulum-associated degradation (ERAD) [99,100,101], mitochondria-associated degradation (MAD) [102] and cytosolic ribosomal protein quality control (RQC) [103]. Moreover, Cdc48 is centrally involved in the tightly controlled chromatin-associated degradation [104] and the engineered Ub fusion degradation pathway (UFD) [105], which proved a useful model system for studying many aspects of Cdc48 in proteasomal degradation. Of note, Cdc48 is not strictly required for the degradation of all substrates of these pathways, as shown, for example, for specific ERAD substrates, for which the proteasomal AAA-ATPases are sufficient for degradation [106,107,108]. In addition to its functions in the UPS, Cdc48 is also involved in other Ub-mediated degradation pathways, for which we refer to [12].

**Figure 4:**
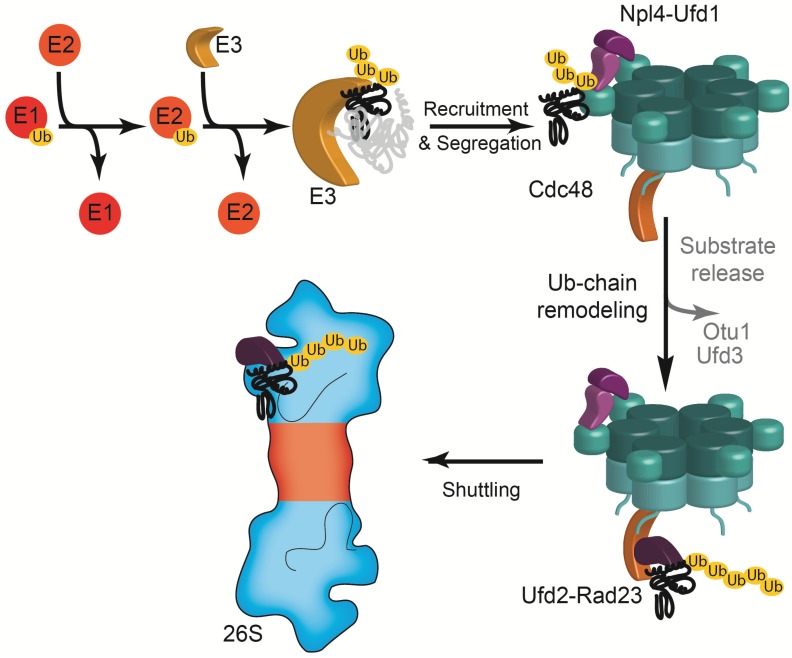
Cdc48-centered escort function for substrates of the UPS. An enzymatic cascade of Ub-activating enzyme (E1), Ub-conjugating enzyme (E2) and Ub ligase (E3) covalently links Ub moieties (yellow) to lysine residues of a protein that is targeted by the UPS. Via the Ub binding Npl4-Ufd1 complex (purple), Cdc48 (cyan) recruits the substrate. The ATP-dependent segregase activity of Cdc48 liberates the substrate from its non-ubiquitylated interaction partners. The fate of the substrate is then determined by Cdc48-associated Ub chain-remodeling cofactors. While the DUB Otu1 and Ufd3 may prolong the lifetime of the substrate by deubiquitylation and substrate release, respectively, the E4 enzyme Ufd2 (orange) extends the poly-Ub chain and facilitates the transfer of the substrate to the 26S proteasome by the shuttling Ub receptors Rad23 and Dsk2 (dark purple).

In contrast to proteasome-associated AAA-ATPases, the major enzymatic function of eukaryotic Cdc48 does not seem to be that of an unfoldase; its main catalytic function is rather that of a “segregase”. Cdc48 segregates ubiquitylated substrates from non-modified partners [109]. These unmodified interactors may be other subunits of a complex if the substrate is part of an assembly, a tightly associated E3 ligase or an organelle membrane. Various cofactors associate with Cdc48 to recruit specific substrates and process them further for degradation. These cofactors tend to be organized in a hierarchical manner and confer specificity to Cdc48 [12]. Here, we only discuss the best-studied examples acting in the UPS (Figure 3 and Figure 4).

Recruitment of the heterodimeric Ub acceptor Npl4-Ufd1 allows Cdc48 to bind to polyubiquitylated substrates [110,111]. Cdc48-associated E4 enzymes, including the RING-related ligase Ufd2 (E4B in mammals), facilitate poly-Ub chain elongation [112]. Similar to the proteasome-associated E4 ligases, the function of Cdc48-associated Ub ligases may be antagonized by Cdc48-associated DUBs, including the ovarian tumor protease Otu1 (YOD1 in mammals) [113], and by Ufd3. The shuttling Ub receptors Rad23 and Dsk2, bind to Ufd2 (PLAA in mammals) [114], enabling substrate targeting to the 26S proteasome (Figure 4). Thus, the functions of PIPs and Cdc48-interacting cofactors appear analogous [115].

## 8. Structure of Cdc48 and Its Associated Machinery

Like the proteasomal AAA-ATPase module and PAN, Cdc48 consists of an N-terminal (N) domain and two ring-forming domains, D1 and D2 (Figure 4). However, in the case of Cdc48, both D1 and D2 are AAA-domains, whereas the rings of the proteasomal ATPases are formed by an OB-domain and an AAA-domain. The structure of the p97 homohexamer has been studied by X-ray crystallography revealing two stacked AAA-rings in head-to-tail orientation and the N-domain tightly-associated with the outer surface of the D1 domain [116,117]. A striking feature of the crystallographic structure is the narrow central pore of the D1 AAA-ring, which is occluded by a pronounced density, possibly a Zn ion. Consistent with this observation, eukaryotic Cdc48, in contrast to its archaeal homologs, lacks critical aromatic residues in the D1 pore that are required for unfoldase activity in ATP-dependent proteases [118]. Thus, the structure and composition of the D1 ring suggest that eukaryotic Cdc48 does not thread substrates through its central channel, in contrast to the proteasomal AAA-ATPases. Consistent with these observations, eukaryotic Cdc48 does not promote the degradation of folded model substrates by the CP, in contrast to its archaeal counterpart [119]. Interestingly, substitution of the eukaryotic D1 pore loop by the archaeal version restores the unfoldase activity of the eukaryotic Cdc48 and according degradation of substrates by the CP [118,119]. Moreover, eukaryotic wild-type Cdc48 promotes the degradation of short peptides, hinting at regulatory function activity [119]. However, this effect is much more pronounced for the archaeal CP than for eukaryotic CP, and it remains to be seen whether the regulatory activity of eukaryotic Cdc48 observed *in vitro* is of physiological importance.

Compared to the lock washer-like quaternary structures of the proteasomal ATPase module, the X-ray crystallographic structures of p97 appear essentially six-fold symmetrical. However, the quaternary structures observed in the crystals are likely to be unphysiological, because they may be induced by packing forces, which are in a similar range as those generated during the nucleotide cycle [117]. For example, the radii of gyration obtained from small-angle X-ray scattering [120] exceed those obtained from the crystal structures for many nucleotide states. Hence, structural insights into the structural rearrangements of Cdc48 during its nucleotide cycle remain on a low-resolution level to date. All nucleotide-binding sites of the D1 ring are occupied with ADP under physiological conditions [121,122], suggesting that the D1 ring functions as a rigid scaffold analogous to the OB-ring of the proteasomal AAA-ATPase module. In contrast, nucleotide binding to D2 is dynamic, with a maximum load of 3–4 nucleotides [121], again similar to CP-associated proteases [9,123]. Upon ATP binding, the D1 and D2 rings rotate with respect to each other by approximately 20° [124,125,126].

The substrate-recruiting cofactors confer specificity and modulate affinities for substrate-processing cofactors, but may also influence the ATPase activity of Cdc48 [127]. To our current knowledge, substrate-recruiting cofactors primarily dock to the N-domain [12]. This domain adopts a pseudo-symmetrical double-psi-beta barrel fold [128,129]. Cofactors bind to the N-domain via Ub regulatory X (UBX) and UBX-like domains, as well as different linear sequence motifs, including the binding site 1 (BS1) and VCP binding (VBM) motifs, often in a competitive manner (reviewed in [130]). The most important substrate-recruiting cofactors, the heterodimeric Npl4-Ufd1 complex and Shp1 (p47 in mammals), which mediates non-proteasomal degradation, bind mutually exclusively to the N-domain.

Both subunits of the Npl4-Ufd1 complex bind to the Cdc48 N-domain, although probably those of neighboring Cdc48 subunits in the holocomplex. The N-terminal Ufd1 truncation 3 domain (UT3) and the Cdc48 N-domain share the same architecture [131,132], whereas the C-terminal domain of Ufd1 is unstructured in isolation and recruited to Cdc48 by its BS1 motif [133]. Both, the Cdc48 and Ufd1 N-domains bind mono- and poly-Ub, suggesting that doubling these domains in a complex is a means of increasing the affinity for ubiquitylated substrates [132]. Npl4 comprises three domains. It binds to the Cdc48 N-domain via its N-terminal UBX-like domain [134]. The central domain of Npl4 is the JAMM-deficient MPN domain, which, analogous to the catalytically inactive MPN domain of Rpn8, has not been assigned to a function at this point. The C-terminal Npl4 zinc finger (NZF) domain, which is only present in higher eukaryotes, binds weakly to free Ub, suggesting that this domain further increases affinity to substrates [135]. Low-resolution cryo-EM data of the glutaraldehyde cross-linked ternary p97-Npl4-Ufd1 complex indicate that one Npl4-Ufd1 heterodimer binds per p97 homohexamer. The holocomplex co-exists in multiple conformations, probably induced by large-scale repositioning of the N-domain dependent on the respective nucleotide state [136].

A major substrate-processing cofactor involved in the UPS is the E4 enzyme Ufd2, which was the first member of this type of Ub ligases specialized for the efficient extension of ubiquitylation nuclei to be identified [137,138,139]. In yeast, Ufd2 binds to the very C-terminus of Cdc48, whereas its mammalian homolog binds to the N-domain via its VBM [137,140]. Ufd2 is antagonized by Ufd3, which binds to the Cdc48 C-terminus through its C-terminal armadillo repeat domain [141,142]. The antagonizing function of Ufd3 is thought to be due to mutually exclusive binding with Ufd2 and/or due to the induction of the release of monoubiquitylated substrates. The DUB Otu1 can bind simultaneously with Ufd3 to Cdc48. It shortens poly-Ub chains and may hence preserve substrate from eventual degradation [113]. Otu1 binds to Cdc48 via its UBX domain that associates with two N-domains, as suggested by X-ray crystallographic analysis [143]. In contrast to proteasome-associated DUBs, Otu1 does not require Cdc48 association for its catalytic activity [113]. The shuttling Ub receptors Rad23 and Dsk2 associate with both 26S proteasome and Cdc48 via Ufd2, enabling the delivery of substrates for degradation. The N-terminal UBL-interacting domain of Ufd2 specifically interacts with high affinity with the UBL domains of Rad23 and Dsk2, but not Ddi1 [114]. This eight-helix domain binds the Rad23 and Dsk2 UBL domains distinct from the modes of interactions seen for Ub-receptors, like Rpn10, Rpn13 and the UBA domains of the shuttling Ub receptors, highlighting the large variation of Ub and UBL binding domains [114]. In summary, the Cdc48 cofactors described here are part of a Cdc48-centered ‘escort function’ for substrates to the 26S proteasome (Figure 4) [112].

## 9. Conclusions

In recent years, structural and functional studies have yielded substantial progress in our mechanistic understanding of proteasomal degradation. In particular, cryo-EM studies have provided us with detailed pictures of the 26S proteasome in its different conformational states, based on which the working hypotheses for the mechanism of degradation could be formulated, which need to be tested now. The forthcoming challenges will be structural studies on the regulation of the 26S by the dynamically-associated PIPs and, eventually, its coordination with Cdc48 and its various cofactors. With the advent of the UPS, Cdc48 seems to have evolved from an AAA-type unfoldase in archaea into a segregase in eukaryotes and acquired a large set of cofactors for substrate recruitment and processing. The large conformational variability of AAA-ATPases makes structural studies of Cdc48 and its cofactors challenging, but such experiments will undoubtedly provide exciting insights into the evolution of this enzyme and the UPS in general.
